# Are nurse`s needs assessment methods robust enough to recognise palliative care needs in people with dementia? A scoping review

**DOI:** 10.1186/s12912-022-00947-6

**Published:** 2022-07-20

**Authors:** Susanne de Wolf-Linder, Margarete Reisinger, Elisabeth Gohles, Emma L. Wolverson, Maria Schubert, Fliss E. M. Murtagh

**Affiliations:** 1 School of Health Science, Institute of Nursing, Zurich University of Applied Sciences, Winterthur, Switzerland; 2 Wolfson Palliative Care Research Centre, Hull York Medical School, University of Hull, Hull, UK; 3 Humber Teaching NHS Foundation Trust, Willerby, UK

**Keywords:** Dementia, Patient Reported Outcome Measure, Nursing Assessment, Needs Assessment, Holistic Nursing, Hospice and Palliative Care Nursing

## Abstract

**Background:**

People with dementia are most at risk of experiencing serious health related suffering, if they do not have a palliative care approach introduced early enough in the illness. It can be challenging for nurses to assess experienced needs of people, who are thought no longer able to self-report such as people with dementia. Assessment help to understand the care the patient and their family need promptly. It is unknown how nurses recognise holistic palliative care needs in people with dementia during routine care.

**Methods:**

Scoping review where EMBASE, MEDLINE, CINAHL, PsycInfo databases, and references were searched with an advanced search strategy, which was built on three concepts (nurses, dementia, and nursing assessment) using corresponding Medical Subject Headings. Data were charted in a piloted extraction form, based on the assessment domains within the nursing process followed by summarise and synthesise results narratively.

**Results:**

37 out of 2,028 qualitative and quantitative articles published between 2000 and 2021, and relating to 2600 + nurses, were identified. Pain was sole focus of assessment in 29 articles, leaving 8 articles to describe assessment of additional needs (e.g., discomfort). Nurses working in a nursing home assess pain and other needs by observing the persons with dementia behaviour during routine care. Nurses in the acute care setting are more likely to assess symptoms with standard assessment tools at admission and evaluate symptoms by observational methods. Across settings, about one third of pain assessments are supported by person-centred pain assessment tools. Assessments were mostly triggered when the person with dementia vocalised discomfort or a change in usual behaviour was observed. Nurses rely on family members and colleagues to gain more information about needs experienced by people with dementia.

**Conclusion:**

There is a scarcity of evidence about techniques and methods used by nurses to assess needs other than pain experienced by people with dementia. A holistic, person-centred screening tool to aid real-time observations at the bedside and used in conversations with health care professionals and families/friends, may improve need recognition other than pain, to ensure holistic needs could then be addressed timely to improve care in people with dementia.

**Supplementary Information:**

The online version contains supplementary material available at 10.1186/s12912-022-00947-6.

## Background

Globally, older people with dementia are most at risk of experiencing serious health related suffering, with a peak predicted in 2060, if they do not receive quality care with a palliative care approach from the onset of dementia [1]. Older people and people with dementia present a challenge to care services: due to their slowly increasing symptom burden [2], they are more dependent on high-quality care provision [3, 4]; however, high quality care can only be provided if symptoms and needs are elicited at the point of care in a systematic way [5] in order to improve quality of life [6, 7].

According to Klapwijk et al. people with multiple illnesses, over 85 years of age, and diagnosed with dementia have the most complex needs [6], extending beyond the elements of the holistic framework (physical, psycho-social, spiritual) of palliative care [8]. Although such experienced needs in people with dementia are often related to quality of life (e.g. being clean and comfortable), others refer to “self-managing dementia symptoms” (e.g. anxiety, wandering, agitation, etc.), need for a friendly and homely environment, and optimal independence [9–11]. Common changes in behaviour in people with dementia [12] pose a considerable challenge to nurses, because of the need to attend to the people with dementia despair, while simultaneously identifying the felt need behind the symptom expressed [13]. To respond to such complexity, it helps nurses to assess symptoms and needs within holistic framework including physical, psychological, social, and spiritual domains [14].

According to national surveys in the UK, good pain and symptom control is the top public concern if living with a life-limiting illness [15], which resonates with care providers elsewhere [16]. Pain has been under-assessed and therefore undertreated in people with dementia [17]. Awareness, knowledge, and practice amongst health care professionals in the recognition of pain could be improved by training and empowering nursing staff to use appropriate assessment tools (e.g. Pain in Advanced Dementia Scale) [18].

Outside specialist palliative care services and depending on the setting, there are different well-developed and validated needs- and symptom-assessment tools for people with dementia available. For instance, the UK National Health Service (NHS) recommends holistic common assessment (including four domains: physical, psychological, social, and spiritual) be undertaken with any person in last year of life, regardless of setting [19]. The assessment is done verbally with the patient and/or with their relatives should the patient no longer be able to provide answers [19]. The Minimal Data Set within the Resident Assessment Instrument (RAI) for people living in the community, as well as RAI-Home (for home care) and RAI-NH (for nursing homes), is completed by a nurse taking a clinical perspective, is available in a number of languages, and used in various countries around the globe [20]. The Minimal Data Set consists of 16 different domains and 238 items to be completed by a qualified nurse together with the patient/resident [21]. For the acute hospital sector, the Comprehensive Geriatric Assessment tool can be used, as it takes a person-centred approach, and is completed by the multidisciplinary team [22].

While these assessment instruments are in use in a variety of settings, it remains unclear whether they can be used to fully elicit and manage people with dementia experienced needs and accompanying symptoms, because i) some instruments are very long and are therefore may not be practical at the bedside [21], ii) others are completed by healthcare professionals, who do not necessarily know the person with dementia well [22, 23], and iii) often only appropriately trained staff can complete the assessment instrument [20, 22], excluding others in the workforce such as for instance nursing assistants [23].

To ensure structured, systematic assessments remain outcome-orientated, i.e. focused on the health status and wellbeing of the person – i.e. ‘person-centred’ [24, 25], nurses must recognise the needs and concerns of people with dementia during care delivery [26, 27]. This is important, because if those observations are documented in a systematic way, the experienced needs of people with dementia might be more readily and easily met at point of care.

Therefore, this scoping review aims to identify how nurses recognise and assess holistic palliative care needs including physical and psycho-social symptoms and other care issues in people with dementia at any time during care provision in the acute-, community-, and nursing home setting. To our knowledge, this will be the first scoping review focused on how holistic needs are assessed during regular care for people with dementia [28, 29].

## Methods

The review answered the following research question:

“Which assessment methods are applied by nurses to recognise unmet, holistic palliative care needs in people with dementia during their regular care?” Objectives derived from the research question were:

- To illustrate which assessment methods are applied by nurses with different levels of education (e.g. registered nurses, nursing aids) and working experience (e.g. in setting or discipline) to assess unmet palliative care needs in people with dementia.
- To describe which assessment techniques are applied by nurses with different levels of education and work experience, leading to holistic need recognition in people with dementia.
- To identify reasons why nurses use a needs assessment tool with people with dementia.
- To report on how nurses identify when to undertake a needs assessment and which assessment tool select for people with dementia.

### Inclusion criteria

#### Definition of needs

In this scoping review we defined’need’ as the “capacity to benefit from health care” [30]. The capacity to benefit from health care encompasses palliative care needs beyond the diagnosis of dementia (e.g. symptom management, provision of comfort, support of family and carers), but remains within the framework of health care [30]. This includes any symptom assessment (e.g. of neuropsychiatric symptoms, delirium, etc.) and conducted at any stage of the disease trajectory including during end-of-life care. Individual needs assessment for people with dementia rests on two pillars from Bradshaw’s taxonomy of need, which are the felt need (what the individual feels) and the normative need (what a professional thinks the person’s with dementia needs are) [31].

#### Definition of research outcomes

For the description of key terms to define research outcomes of this review [32], the Population, Exposure, Outcome of interest, and Study type framework for was used [33]. Nurses were considered to be the main population (P) in this review. Included were nurses with any educational level (registered nurses (RN), licensed practice nurses (LPN), health care assistants (HCAs), and nursing aids), conducting a needs assessment including measures or tools used, and related prompts or triggers for conducting assessment of people with dementia or people with dementia related cognitive impairment. The exposure (E) was assessment of a person with a diagnosis of dementia or dementia related cognitive impairment or mild cognitive impairment, and the outcome (O) was how needs are recognised and assessed. A needs assessment was defined as a method and/or assessment or outcome tool, which allows nurses to assess needs regularly in order to plan, conduct, and monitor care in a person-centred way [34]. The outcomes of interest are the different types of assessment methods conducted, including corresponding prompts or triggers for assessment of needs, and evaluation of previous assessments.

### Types of participants

#### Definition of population: nurses (conducting the needs assessment)

The study population for this review were all nurses. Nurses with a tertiary qualification are educated and trained to take on specialist-, management-, triage-, research-, and / or teaching roles, often managing or teaching nurses trained to secondary or primary level [35]. This group is less likely to care for people in primary care and nursing homes on the frontline [35, 36]. In contrast, nurses, with a secondary qualification are widely known as health care professionals and often work at the frontline and manage the workload amongst the team [37]. Nurses with a primary qualification are mostly auxiliary staff and nurse assistants, who are working under supervision of a secondary or tertiary qualification nurse [38]. For the purpose of this review, the nurses’ professional categories (RNs, LPNs, nursing aids, HCAs) were used as they represent their educational preparation (e.g. Diploma Nurse) and their work experience in years worked as a nurse and/or current setting [39].

#### Definition of exposure: assessment of people with dementia

The exposure was assessment of people with dementia (people diagnosed with dementia and/or people with suspected dementia) at any point during their disease trajectory, who were assessed in their usual place of residence, a nursing home, or an acute hospital.

#### Definition of outcome (recognition and assessment of holistic needs)

Holistic-needs-Assessment:

A standardised clinical needs assessment (e.g. Comprehensive Geriatric Assessment, Holistic Common Assessment), conducted by a health care professional is key to eliciting patients’ needs [19, 40]. Such an assessment is designed to help understand and co-ordinate the care of the patients and their family need promptly, by initiating tailored interventions [19, 40, 41]. The method of the initial assessment is formal and usually recorded in a patients care record [19, 20]. However, conducting a needs assessment and in particular a repeated assessment in someone with dementia who is likely to have multiple chronic health problems may not be straightforward [42]. As this review focused on how needs are recognised and assessed in a range of settings, various methods of and triggers for recognition and assessment of needs were charted for analysis, including documented assessment or evaluation of needs (verbal or non-verbal) at any time point during care (e.g. during helping the patient having a bath) or via telephone or other media (e.g. Skype).

Study designs included were peer-reviewed original research with both qualitative and quantitative research paradigms. Studies where nurses were the biggest group of participants carrying out the assessment and reported in results, accordingly, were also included to avoid losing important research findings relevant for this review.

### Exclusion criteria

Because this review focused on the nursing population, studies in which assessments were carried out by groups other than nurses (e.g., doctors, volunteers) were excluded. Articles, in which patients with psychotic disorders, such as forms of schizophrenia and bipolar illnesses, and older people with mental health and substance abuse issues (which can cause dementia) were excluded from this review because this review focused on the needs assessment in people who are suffering cognitive impairment due to a diagnosis of dementia and not due to other causes. Articles were excluded if they were clinical case reports, systematic reviews, letters, and editorials as no interpreted research findings could be untangled in those designs. Articles reporting intervention programmes and -trials were excluded as they don’t currently mirror the day-to-day needs assessment of most front-line nurses caring for people with dementia. Articles in languages other than English and German were also excluded due to limited language resources within the research team.

### Search strategy

The search approach adopted search strategies used in published protocols and was designed to capture the key terms as broadly as possible [43]. The search strategy was trialed in a pilot exercise to identify its sensitivity and specificity within different databases before they were adjusted for each database.

Medical subject headings (MeSH) terms used in the finalized strategy were: ‘nurses’ or ‘nurse practitioner’ AND ‘Dementia’ OR ‘cognition disorders’ OR ‘Alzheimers disease’ or ‘cognitive dysfunction’ or ‘memory disorders’ AND ‘nursing assessment’ OR ‘Symptom Assessment’ OR ‘Needs assessment’.

### Electronic databases

For this scoping review, the databases below were searched from the year 2000 onwards [44]. Earlier studies were not considered, because the tertiarization of the nursing profession was introduced in most European countries around the year 2000 [45]. The Nursing and Midwifery Council, a regulatory body overseeing clinical practice skills, performance, misconduct, and education, brought on a critical change in the nursing profession in the United Kingdom (UK), also in terms of new role definitions, which, it was felt, warranted the range of years (2000-present) chosen for this review [46]:

- Medline(R) via Ovid In-Process & Other Non-Indexed Citations and Medline (R) via Ovid (2000 to present)
- PsycINFO (2000 to present)
- CINAHL via EBSCO (2000 to present)
- EMBASE (accessed on:)
- Reference search

### Supplementary searching

Following the initial search in the databases, and identification of eligible studies, a thorough reference search was conducted. Systematic reviews were excluded because of the complexity to tease out findings for this review. But systematic reviews were first included and used for reference check before excluding them again [18, 28, 29, 47–57].

### Selection of studies

As soon as the scoping review protocol including search string was approved in June 2020 by the research team, the initial search was run with an updated search in May 2021 to capture all citations. All references were identified and exported to the bibliographic program EndNote and screened in title and abstract against in- and exclusion criteria as described above. After duplicates were removed by the reviewer author, full texts of all relevant studies were retrieved, read, and selected using the same procedure.

A second reviewer assessed one quarter of the retrieved references, blinded to the author reviewer’s selection. Differences in the choice of articles for inclusion were discussed and resolved by consensus. A flow diagram of study selection is illustrated in line with the PRISMA-extension for scoping reviews criteria [58] in the results section.

### Protocol and registration

This scoping review is based on the corresponding scoping review protocol, which can be requested from the authors of this paper.

### Charting the results

First, an overview of included studies (Title of study, authors, year, aim of the study/purpose, study design, population/sample size, journal, country, results) was charted in an Excel sheet to provide a summary of the included evidence.

Data were charted using the internationally approved nursing documentation framework Subjective, Objective, Assessment, and Plan to record patients’ health concerns [59, 60]. Within this framework data from articles were charted into the following sections of the piloted data extraction sheet in Excel:

- Setting
- Patient population
- Nurse’s professional category of nurse completing the assessment
- Familiarity with patient/resident
- Advice/Information received by whom in order to complete the Assessment/outcome
- Reason/Triggers for nursing assessment
- Time point of assessment/measurement
- Name of need(s), which was/were assessed
- How was the experienced need assessed
- Severity of experienced need assessed
- Type of Assessment/Measurement (PROMs, Proxy, Nurse led)
- Name of Assessment-/ Outcome measure
- Other benefits of the assessment / measurements (knock-on effect for the patient, patient care, nurses, planning, etc.)

The results were then summarised and synthesised narratively adhering to the concurrent structure of the objectives and the review’s research question.

## Results

### Results I – study characteristics:

**Fig. 1:**
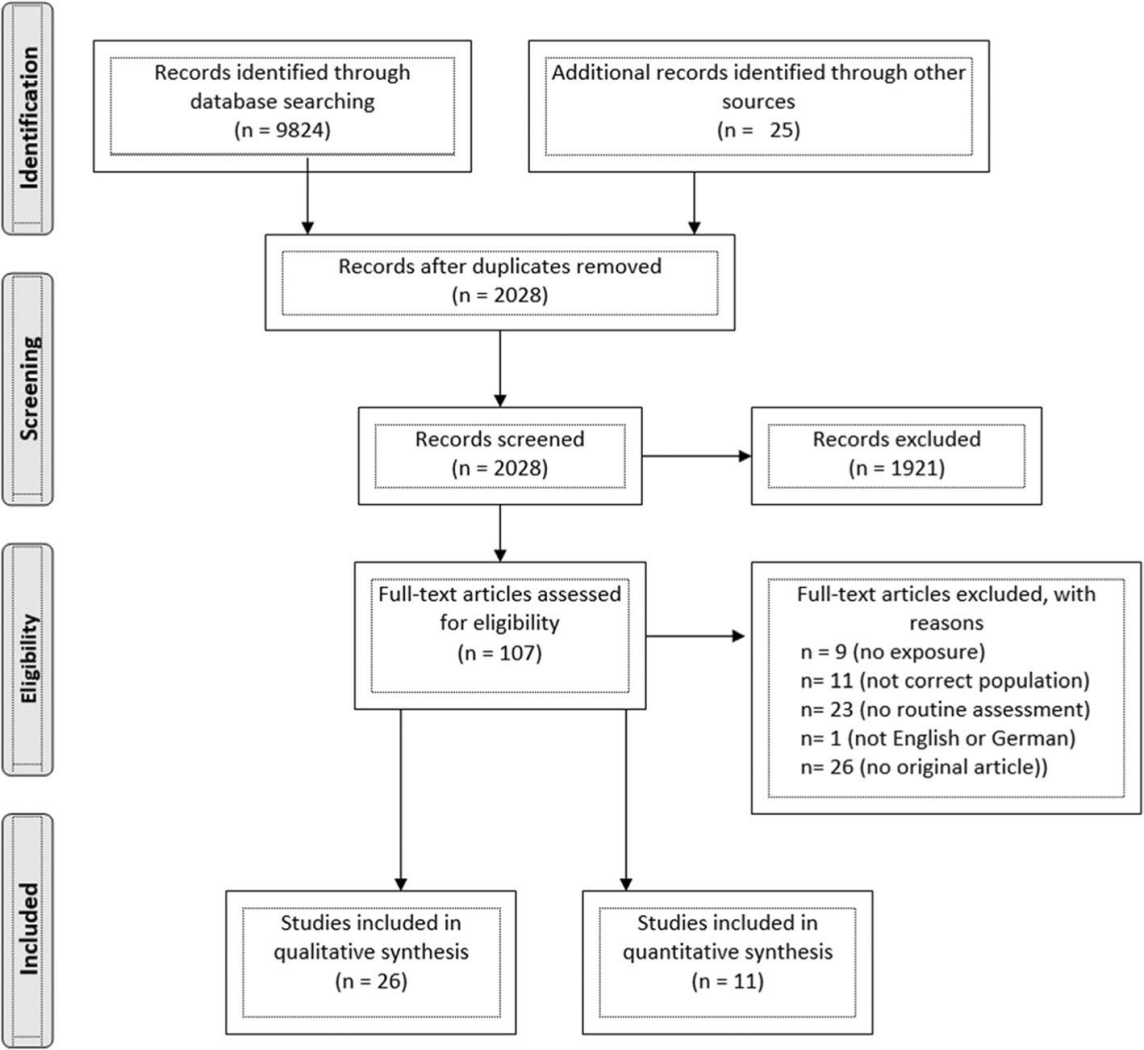
PRISMA flow diagram of study selection and inclusion process

In total, 9,824 studies were identified. 7,796 studies were removed, as they were duplicates, published prior to the year 2000 or published in a language other than English or German. 107 studies were retrieved for full text screening after excluding another 1,921 publications due to not meeting inclusion criteria. Please refer to Fig. 1 for more detailed information on the study selection process.

#### Study characteristics of included studies

Eleven studies with a quantitative [61–71] and twenty-six studies with a qualitative [72–97] research paradigm were included. However, there was a wide range of research methods and data collection approaches used, ranging from action research approaches, to interviews, to ethnographic accounts, questionnaires, and extensive chart reviews and audits. The year of publication ranged from 2001 [72] to 2021 [66, 93], with most studies having been published between 2011 and 2021. However, the aims amongst the studies remained within similar scope over the years (Table 1).

**Table 1:** Study characteristics overview of included studies

Title of the study	Authors, Year, Country	Aim of the study / Purpose	Population, Sample Size	Journal	Results relevant to the review’s research question
An exploration of pain documentation for people living with dementia in aged care services	Andrews S.M. et al., 2019 Australia [61]	To assess the quality and completeness of pain documentation by nurses	People with moderate to severe dementia: *n* = 114 Pain episodes: *n* = 169	Pain Management Nursing. IF: 1.929	• One pain episode was documented in 86% of residents • 29% of pain episodes had no documentation on how nurses identified pain in People with dementia • In *n* = 120 People with dementia pain identification was documented using no assessment tools • Personal care assistants were responsible for more episodes of assessment documented compared with nursing staff (50% vs. 18%)
An exploration of nursing home managers` knowledge of and attitude towards the management of pain in residents with dementia	Barry, H.E. et al., 2012 United Kingdom / Ireland [62]	To explore the knowledge, attitudes and beliefs that nursing home managers hold regarding pain assessment, in People with dementia	Nursing home managers: *n* = 96	International Journal of Geriatric Psychiatry. IF: 2.419	• Nursing home managers share different opinions with regards to how assess and manage pain in People with dementia • Barriers in the assessment of pain are: missing report about pain from the People with dementia, lack of time to assess pain in People with dementia, and lack of standardised approach to treat pain
Recognising pain in older adults living in sheltered accommodation: the views of nurses and older adults	Blomqvist K. and Hallberg I.R. 2001 Sweden [72]	To illustrate nurses and older adults views about how to recognise the presence of pain in older adults with dementia living in special needs housing	Contact nurses and Older adults with frequent pain and cognitive impairment: *n* = 24	International Journal of Nursing Studies. IF: 3.783	• 24 of 42 cognitively impaired people were often in pain • 16 of 24 were in pain every day • pain recognition is a communicative interactive process based on verbal and non-verbal expressions
Pain relief at the end of life: Nurses’ experiences regarding end-of-life pain relief in Patients with dementia	Brorson, H. et al., 2014 Sweden [73]	To describe nurses' experiences regarding end-of-life pain relief in patients with dementia	Nurses: *n* = 7 with no formal palliative care or pain management training in People with dementia	Pain Management Nursing. IF: 1.929	• Nurses' experiences of resources concerning pain relief with the subcategories: ability to understand the needs of the patient, interpersonal relationships, cooperation centring on the individual, feeling of satisfaction to relieve suffering with pharmacological pain relief options, and feeling of satisfaction to relieve suffering with nonpharmacological pain relief options
Challenges for professional care of advanced dementia	Chang, E. et al., 2009 Australia [74]	To expose the challenges for key professional providers of care for people with advanced dementia living in residential age care facilities	Nurses: *n* = 24 (Director of nursing *n* = 2; Registerd nurses from different specialities = 15; nurses = 2, Nursing assistants = 5	International Journal of Nursing Practice. IF: 1.133	• Challenges for key professional carers: identified main areas: Key professional carers felt 1) Lack of knowledge and skills in the direct provision of care with regards to symptom management; 2) lack of knowledge relating to dementia as a disease; 3) Lack of knowledge relating to palliative care • Participants emphasized the need to improve knowledge and skills and the need for policy changes
Concept analysis of nurses' identification of pain in demented patients in a nursing home: development of a hybrid model	Chang, S. et al., 2011 Korea [75]	To clarify and conceptualize the phenomena of pain identification in nurses caring for People with dementia	Nurses (> 3 years nursing home experience): *n* = 13	Pain Management Nursing. IF: 1.929	• Nurses identified and managed pain in the constant process of nursing: 1) an active process integrating every expressional cue of patients until pain relief was achieved, 2) a cyclic process that begins upon a patient's expression of pain 3) an active process of comparison on patient's usual expressive patterns
Pain Assessment Practices with Nursing Home Residents	Clark, L. et al., 2004 USA [76]	To determine how nursing home staff currently think about and conduct resident pain assessments	Focus groups with 3 to 20 participants per group (Registered nurses*, Certified nursing assistants*, and allied health care professionals): *n* = 20 *Only data from Registered nurses and Certified nursing asstistants charted	Western Journal of Nursing Research. IF: 1.217	• Uncertainty in pain assessment • Relationship-centred cues to residents' pain • Behavioural and visual cues to residents' pain • Complications of resident characteristics and attitudes in the accurate assessment of pain • Relationship to the People with dementia as well as staff knowledge of residents' usual behaviour process were in most cases the foundation in the identification of pain
Cues for the identification of pain in nursing home residents	Closs, S. et al., 2005 United Kingdom / England [77]	To identify a range of cues from which pain was understood by nurses for residents with various levels of cognitive impairment	Nursing home staff: *n* = 65 and informal carers (relatives and friends): *n* = 36 of 113 residents from *n* = 15 UK nursing homes	International Journal of Nursing Studies. IF: 3.783	• Verbal or body language cues • Acute behavioural cues, and general changes in behaviour or mood • Body movements were the most frequently used indicator of pain and were increasingly used as guidance when cognition deteriorated
Nursing Staff Members' Perceptions of Pain Indicators in Persons with Severe Dementia	Cohen-Mansfield J. and Creedon, M. 2002 USA [78]	To assess nursing staff members' perceptions of behavioural expression of pain	Nursing staff: *n* = 29 (Nurse managers: *n* = 7; Charge nurses: *n* = 7; Nursing Assistants: *n* = 15)	The Clinical Journal of Pain. IF: 2.893	• Behavioural expression of pain such as facial grimacing = 76% • Increased agitation as indication of pain = 49% • Observation of the People with dementia by touching a particular body part = 45% • Sudden limping, changes in vital signs, and falls are to the evidence new clues to assess pain in People with dementia
Assessment and treatment of behaviour problems in dementia in nursing home residents: a comparison of the approaches of physicians, psychologists, and nurse practitioners	Cohen-Mansfield, J. et al., 2012 USA [63]	To compare physicians, psychologists, and nurse practitioners approaches to the assessment and treatment of dementia-related behaviour problems in nursing home residents	Total participants: *n* = 246 Medical doctors: *n* = 108; Psychologists: *n* = 38; Nurse practitioners: *n* = 100. *Only data from Nurse practitioners charted	International Journal of Geriatric Psychiatry. IF: 3.485	• Resisting care and disruptive vocal/verbal behaviours were the most common symptoms noted by all professions • NPs were significantly more likely to speak with family and to observe the resident outside of the formal assessment
Exploring healthcare assistants’ role and experience in pain assessment and management for people with advanced dementia towards the end of life: a qualitative study	De Witt Jansen, B. et al., 2017 United Kingdom / Ireland [81]	To explore Health care assistants perspectives and experiences of pain assessment and management in people with advanced dementia approaching the end of life in hospice, acute care and nursing home settings	Total: *n* = 14; Health care assistants from Nursing home: *n* = 9; Health care assistants from acute care: *n* = 2; Health care assistants from hospice: *n* = 3	BMC Palliative Care. IF: 2.015	• Recognising pain (knowing the resident, observing and interpreting behavioural and nonverbal indicators of pain) • Reporting pain (positive work-related identities and relationships, negative work-related identities and relationships) • Training and upskilling
Emergency nurses evaluation of observational pain assessment tools for older people with cognitive impairment	Fry, M. et al., 2017 Australia [79]	To identify emergency departments nurses’ perceptions of the feasibility of Pain Assessment in Advanced Dementia Scale to assess, monitor and manage pain in people over 65 with Cognitive Impairment and the utility of Pain Assessment in Advanced Dementia Scale when compared to other pain assessment observational tools (the Abbey Pain Scale, Doloplus-2 and PACSLAC-Scale)	Nurses: *n* = 36 (total); Focus-groups: *n* = 6 (in three different hospitals)	Journal of Clinical Nursing. IF: 3.036	• PAINAD gives structure to pain assessment • PAINAD assists to convoy pain intensity • Pain assessment tools, such as PAINAD tool, have the potential to achieve greater consistency within ED practice with minimal impact on workload
Understanding Nurses Decisions to Treat Pain in Nursing Home Residents with Dementia	Gilmore-Bykovskyi A.L. and. Bowers B.J 2013 USA [80]	To examine nurse’s decision-making to pharmacologically treat pain in People with dementia living in a nursing home	Nurses: *n* = 13 (Registered nurses: *n* = 10; Licenced practice nurse: *n* = 3)	Research in Gerontological Nursing. IF: 1.571	• Nurses perceived level of certainty about the presence of pain in People with dementia with the most significant factor in determining whether and how quickly resident`s pain would be treated pharmacologically • The developed concept revealed different definitions of pain indicators such as behavioural, visible, non-visible, and self-report
Evidenced based assessment of acute pain in older adults	Herr, K. et al., 2004 USA [64]	To report baseline and pre-intervention data from medical records of patients (> 65 years) and from questionnaires regarding pain management practices, completed by nurses. *only data from People with dementia were used	Patients: *n* = 709 (with dementia: *n* = 181) from *n* = 12 acute geriatric settings. Questionnaires completed by nurses: *n* = 172	The Clinical Journal of Pain. IF: 2.893	• People with dementia had a higher percentage of pain assessment every four hours over the 72-h period • People with dementia had fewer reassessment within 60 min in the entire 72-period • People with dementia had at least one assessment of pain intensity using a rating scale (*P* < .0001) • Most nurses (93.6%) believe that the use of a pain rating scale is the preferred practice, but only 41.9% are using a pain rating scale on a regular basis; • The greatest challenge for nurses to assess pain was difficulty in communicating with People with dementia
Registered Nurses’ View of Performing Pain Assessment among Persons with Dementia as Consultant Advisors	Karlsson, C. et al., 2012 Sweden [82]	To illustrate registered nurses` view of pain assessment in People with dementia in relation to their nursing profession role as consultant advisor	Registered Nurses: *n* = 11	The Open Nursing Journal IF: 1.363	• Four categories were identified amongst RNs with regards to their view of pain assessment in their role as nurse consultants: 1) Estrangement from practical nursing care (e.g., by feeling of remoteness from patient) 2) Time consuming and unsafe pain documentation (e.g. being a second-hand receiver of pain information) 3) unfulfilled needs of reflection possibilities (e.g. Being in a supervisor role)
Certified nursing assistants' perception of pain in people with dementia: a hermeneutic enquiry in dementia care practice	Karlsson, C. et al., 2013 Sweden [83]	To interpret certified nursing assistants' perception of pain in people with dementia in nursing care practice	Certified nursing assistants: *n* = 12	Journal of Clinical Nursing. IF: 3.036	• CNAs perspective origins in being close and familiar with the People with dementia rather than referring to medical knowledge • Perceptions arise in three several phases in CNAs: being in the facing phase (e.g. Observing expressions of pain), being in a reflective phase (e.g. comparing a People with dementias behaviour with his/her behaviour from recent days), and being in an acting phase (e.g. examining physical sign of pain)
Home healthcare teams' assessments of pain care recipients living with dementia: a Swedish exploratory study	Karlsson, C. et al., 2015 Sweden [84]	To explore home healthcare teams' experiences of pain assessment among care recipients with dementia	Nurses: *n* = 23 (total); Registered nurses: *n* = 13; Certified nursing assistants: *n* = 10	International Journal of Older People Nursing. IF: 2.115	• Four interventions, which describe home healthcare teams' experiences of assessing pain were developed: The need for trusting collaboration, the use of multiple assessment strategies, maintenance of staff continuity in care and assessment situations, and the need for extended time to assess pain
Caring for cognitively impaired nursing home residents with pain	Kenefick A.L. . 2004 England [85]	To describe beliefs and behaviours of registered nurses regarding the assessment of pain in cognitive impaired nursing home residents	Nurse key informants (head nurses): *n* = 3	International Journal for Human Caring. NK	• Nurses were more concerned with the characteristics of the nurse than they were with characteristics of the resident to identify and assess pain in People with dementia • Personal knowledge of the resident and nurse-resident interaction was essential to the identification of the resident with pain • Ability to identify important aspects of a resident's behaviour was improved by knowing the individual resident and their experience in geriatric nursing
Decision-making in caring for people with dementia at the end of life in nursing homes	Koppitz, A. et al., 2016 Switzerland [86]	To understand nurses' decision-making process about symptom management for people with dementia in nursing homes in their terminal phase of life	Nursing home staff (Registered nurses, Health care assistants, Nursing aids): *n* = 32	International Journal of Palliative Nursing. IF: 0.72	• Factors influencing nurses' decision-making-process: infrastructure; time allocated to individual residents; the attitude of the nurses and their managers towards the quality of care of People with dementia in order for nurses to reflect with other HCPs and informal carers involved in the care of the person with dementia
Nurses' perceptions of pain assessment and treatment in the cognitively impaired elderly: It's not a guessing game	Kovach, C. R. et al., 2000 USA [87]	To describe nurses' perceptions regarding the assessment and treatment of pain in patients with late-stage dementia	Nurses for single interviews (Licenced practice nurses = 53%; Registered nurses = 47%): *n* = 30; Care nurses in focus-group for rating: *n* = 16	Clinical Nurse Specialist. IF: 1.067	• Signs and symptoms of pain: facial grimacing and restless body movements were the most common signs described • Decreased appetite and combative behaviour were other common symptoms • Pain medication should more often be used as the first pharmacologic intervention to treat behavioural symptoms
Experience of registered nurses in assessing postoperative pain in hip fracture patients with dementia	Krupic, F. et al., 2018 Sweden / Bosnia Herzegovina [90]	To explore the experience of registered nurses in assessing pain in hip fracture in patients with dementia in the postoperative setting	Registered nurses: *n* = 51	Medicinski Glasnik. IF: 0.99	• Nurses' factors that influence their assessment of pain in People with dementia after surgery are illustrated in two main categories (visual assessment and communication) • Seven subcategories (facial expression, body language, behavioural changes, being informed about dementia patients, communication about pain, reporting pain, suggestions for improvement) underpinning a visual than a communicative approach to assess pain
Experience of nurses in assessing postoperative pain in hip fracture patients suffering from dementia in nursing homes	Krupic, F.et al., 2020a Sweden / Bosnia Herzegovina [88]	To explore the experience of registered nurses in the assessment of pain and communication with hip fracture patients with dementia in nursing homes	Registered nurses: *n* = 24	Medicinski Glasnik. IF: 0.99	• Nurses approach pain assessment of a post-surgical People with dementia after hip-fracture by communicating verbally, then by using non-verbal communication (e.g. touching the People with dementia), before doing a pain assessment using a holistic approach • Nurses felt scope for improvement (e.g. more time available) to be helpful in the assessment of pain
Experience of Intensive Care Nurses in Assessment of Postoperative Pain in Patients with Hip Fracture and Dementia	Krupic, F. et al., 2020b Bosnia Herzegovina/ Sweden [89]	To explore the experience of intensive care nurse’s assessment of pain in patients with hip fracture and dementia in the post-operative setting	Intensive care nurses: *n* = 21	Mater Sociomed IF: NK	• Nurses assess pain in People with dementia after surgery of a hip-fracture by communicating with the People with dementia before looking out for visual signs of pain (e.g. expression of pain through body language) • Lack of knowledge about the People with dementia influence pain assessment
Concept Development of Identification of Discomfort for Nursing Home Patients with Advanced Dementia	Lee, S.J. et al., 2020 Korea [91]	To facilitate the assessment of discomfort in People with dementia, living in a nursing home, by nurses and caregivers for concept development purposes	For fieldwork: *Nurses: *n* = 12; *Care helpers: *n* = 8; Physiotherapist: *n* = 5; Social workers: *n* = 5; Occupational therapist: *n* = 1. *Only data from Nurses and care helpers charted	International Journal of Nursing Knowledge. IF: 1.222	• Identification of discomfort is a complex phenomen because of the People with dementias indirect expression of discomfort (e.g. by getting agitated) • Nurse`s interactive and confirmative process of sympathetically diagnosing a People with dementias unmet needs by taking a holistic approach involving other caregivers into the process
The assessment and management of pain in patients with dementia in hospital settings: a multi-case exploratory study from a decision-making perspective	Lichtner, V. et al., 2016 England, Scotland, USA [92]	To understand how pain is recognised, assessed, and managed in patients with dementia by health care professionals in acute hospital settings	Interviews: Health care professionals: *n* = 52 (*Health care assistants: *n* = 7; *Nurses: *n* = 31; Physicians: *n* = 3; Medical consultants: *n* = 5; Pharmacist: *n* = 1; Physiotherapiests: *n* = 1; Clinical educators: *n* = 4); Informal carers: *n* = 4 *Only data from Health care assistants and Nurses charted	BMC Health Services Research. IF: 1.987	• In order to recognise, assess, and manage pain in People with dementia in the acute hospital setting, various information sources and individuals at different times and in different places need to be present • Four over-arching themes identified during interviews to understand how pain in People with dementia was assessed: communication pain with dementia, carer-clinician communications, trials with therapy, putting a picture together
End-of-life care for people with advanced dementia and pain: a qualitative study in Swedish nursing homes	Lundin E. and Godskesen T.E. 2021 Sweden [93]	To describe the experiences of pain management of nurses in caring for people with advanced dementia and pain at the end of life	Nurses: *n* = 13 (Registered nurses = 11; nurses = 2)	BMC Nursing. IF: 2.59	• Challenges in terms of assessing and relieving pain (communication) such as uncertainties when to assess pain • The influence of relatives (relational), and time constraints due to various reasons (organisational) were described by nurses caring for People with dementia
Complexities of pain assessment and management in hospitalised older people: A qualitative observation and interview study	Manias E. 2012 Australia [94]	To examine how pain was assessed and managed in older patients who were admitted to geriatric evaluation and management units	Sample Geriatric patients: *n* = 285 including *n* = 15 patients with cognitive impairment; Registered nurses: *n* = 34	International Journal of Nursing Science. IF: 2.62	• Insight into how nurses communicate with each other and with older patients about pain assessment and management in geriatric evaluation and management units—four major themes were identified: communicating among nurses and between older patients and nurses; strategies for pain management; environmental and organisational aspects of care. *from this study, one paragraph explaining about physical observation of pain in People with dementia was extracted for the review
Assessment of pain in cognitively impaired (CI) older adults in long-term care	Mezinskis, P. M. et al., 2004 USA [65]	To examine formal and informal methods of pain assessment nurses and caregivers use for cognitively impaired people	Long term care facilities: *n* = 14. Nurses: *n* = 160 direct caregivers (*n* = 35 Registered nurses; *n* = 41 Licenced practice nurses; *n* = 81 Certified nursing assistants). Residents: *n* = 307 Cognitively impaired older adults with chronic painful illness	Geriatric Nursing. IF: 2.361	• About 60% of RNs used formal pain assessment tools together with informal pain assessment methods (e.g. Change in usual behaviour)
Pain management nursing practice assessment in older adults with dementia	Minaya-Freire, A. et al., 2021 Spain [65]	To assess pain management nursing practice in older adults with dementia through electronic health records	Electronic health record review from 18 Registered nurses on a 24-bed ward People with dementia included: *n* = 111	Nursing Open. IF: 1.363	• Pain intensity was assessed a median of 1.9 times per day of stay, more of the assessments were made during late shift (39%)
Decision factors nurses use to assess pain in nursing home residents with dementia	Monroe, T. B. et al., 2015 USA [95]	To assess nurses cues and practices to identify and alleviate pain in People with dementia, living in the nursing home	Nurses (not specified): *n* = 29; four focus groups from two facilities	Archives of Psychiatric Nursing. IF: 1.266	• In identifying pain, nurses felt to "put together" a complex jigsaw puzzle to elevate the People with dementias pain • Five subthemes were identified: 1) Uncertainty about the pain experience of residents with dementia, 2) being a detective, 3) clarifying factors, 4) conflict resolution (balancing family wishes and resident's needs), 5) role of the nurse
E-Learning course for nurses on pain in patients unable to self-report	Muñoz-Narbona, L. et al., 2020 Spain [67]	To assess knowledge on pain assessment in nurses following an online training course. *For this study, only data from the pre-course assessment were used	Nurses (not specified): *n* = 401	Nurse Education in Practice; IF: 2.281	• 37.7% reported having no specific training on pain and just 32.2% reported using the PAINAD-Sp scale; 32.2.% was unable to specify the method used to assess pain, 19.4% are discussing the pain assessment with the physician and 16.2% were consulting relatives
Silent and suffering: a pilot study exploring gaps between theory and practice in pain management for people with severe dementia in residential aged care facilities	Peisah, C. et al., 2014 Australia [96]	To explore attitudes and processes relating to pain assessment and management for people with severe dementia in residential aged care facilities	Nurses: *n* = 20 (*n* = 4 care managers; *n* = 10 Registered nurses; *n* = 6 personal care workers. Residential aged care facility: *n* = 15	Clinical Interventions in Aging. IF: 4.458	• All facilities use Abbey Pain Charts and two the Pain Assessment in Advanced Dementia Scale • Time and Frequency of assessment was regulatory-driven • Seven out of 20 reported behaviour changes as trigger for pain assessment, CNAs detect pain—RNs initiate treatment
Communication and assessment of pain in hip fracture patients with dementia—experiences of healthcare professionals at an accident and emergency department in Sweden	Seffo, N. et al., 2020 Sweden [97]	To describe the experience of healthcare professionals in assessing pain and communication in patients with hip fractures and dementia on an ED (emergency department)	Nurses: *n* = 21 (*n* = 17 Registered nurses; *n* = 4 Nurse assistants)	Medicinski Glasnik. IF: 0.99	• Three main categories identified where assessment and communication is time pressured: 1) arrival at the emergency department: patients with dementia cannot be left alone in a room without supervision, someone has to be ready to intervene quickly and the interaction and communication is important, 2) hip track: patient with fracture and dementia should not stay at ED, and instead go to the ward immediately 3) handover to the ward: often the ward is unable to receive patients
Pain Assessment and Dementia—German Results from a European Survey	Sirsch, E. et al., 2015 Germany, Austria, Switzerland [68]	To develop a toolkit for pain assessment in people with cognitive impairment	Nurses (with range of qualifications and specialities): *n* = 209 Others: *n* = 6	Pflege und Gesellschaft—Zeitschrift für Pflegewissenschaft. IF: NK	• Pain assessment is performed almost exclusively by nurses, 44% of respondents do not use guidelines and standards • 52% do not use a standardized observation tool to assess pain • Uncertainty, lack of objectivity, lack of time, and lack of knowledge contribute to barriers of pain assessment
Nurse recognition of delirium superimposed on dementia in hospitalized older adults	Steis, M.R., 2009 USA [69]	To explore acute care nurses' recognition of delirium in hospitalized older adults with dementia	Review of nursing records (*n* = 108) *n* = 140 hospitalized older adults with dementia	Dissertation in Nursing. IF: NA	• The most significant finding from this study was the absence of patient days on which nurses recognized delirium in this population of hospitalized older adults with dementia • Nurses more likely to document disturbance of consciousness
Acute hospital dementia care: results from a national audit	Timmons, S.et al., 2016 Ireland [70]	To assess the quality of dementia care in acute hospitals in the Republic of Ireland	Acute public hospitals (*n* = 35) tracking the quality of care received by *n* = 660 patients. Interviews with *n* = 76 ward managers	BioMed Central Geriatrics. IF: 3.077	• Pain and functioning is underassessed in people with dementia • Assessment for cognition (43%) and delirium (30%) was inadequate
Pain in older adults with dementia: A survey across Europe on current practices, use of assessment tools, guidelines and policies	Zwakhalen, S. et al., 2018 Austria, Belgium, Denmark, Germany, Netherlands, Switzerland, United Kingdom [71]	To explore the Health care professional`s use of existing tools to assess pain and to identify attitudes towards assessment tools in older adults with cognitive impairment	Health care professionals and care assistants: (*n* = 810; *n* = 206 HCP in hospital care; *n* = 127 long term care; *n* = 38 primary care; *n* = 439 unknown setting/	Der Schmerz; IF: 1.107	• Less than half of healthcare staff are using specific pain assessment tools • Most health care professionals do not feel competent enough in pain assessment. Most pain assessment is done during routine care

Most studies stemmed from Europe [62, 66–68, 70–73, 77, 81–86, 88–90, 92, 93, 97] and were conducted in nursing homes [61–63, 65, 68, 71, 72, 74–78, 80–83, 85–88, 91, 93, 95, 96] (Table 2). However, the sample, as illustrated in Table 2, was diverse in terms of continent and settings in which the studies were conducted. The sample was less diverse in terms of needs assessed, as pain dominated the sample as the main symptom assessed in 28 studies [61, 62, 64–68, 71–73, 75–77, 79–82, 84–86, 89, 90, 92–97]. Only discomfort [91], delirium [69], and behaviour [63] other than pain were assessed in addition, in six studies [70, 74, 78, 83, 87, 88].

**Table 2:** Study characteristics summary

**Origin of studies (n)**		**Reference**
North-America	9	[63–65, 69, 76, 78, 80, 87, 95]
Australia	5	[61, 74, 79, 94, 96]
Europe	21	[62, 66–68, 70–73, 77, 81–86, 88–90, 92, 93, 97]
Asia	2	[75, 91]
**Population summarised from all studies included (n)**		
Nurses (Registered Nurses, Health care assistants, Nursing aids)	2600 +	[61–97]
**Number of studies reporting on setting (n)**^a^		
Nursing home	24	[61–63, 65, 68, 71, 72, 74–78, 80–83, 85–88, 91, 93, 95, 96]
Community setting	3	[72, 82, 84]
Acute care	12	[64, 66–71, 73, 81, 90, 92, 94]
Hospice	1	[81]
Emergency department	2	[79, 97]
Intensive care unit	1	[89]
**Number of studies assessing needs (n)**		
Pain	28	[61, 62, 64–68, 71–73, 75–77, 79–82, 84–86, 89, 90, 92–97]
Pain and other	6	[70, 74, 78, 83, 87, 88]
Other Needs (Discomfort, Delirium, Behaviour)	3	[63, 69, 91]

^a^more than one setting possible

More than 2,600 nurses were included in these studies. Numbers are likely even higher, because some authors did not report on the number of nurses they included in their studies. Most studies included RNs hence these form the biggest part of the population of this scoping review. However, at least 13 out of 37 studies of studies also included health care professionals (HCP) and/or LPNs, and/or nursing aids. Only one study reported on views from nurse ward managers and one study reported views from nurse practitioners.

### Result II – nurses’ assessment methods to recognise unmet palliative care needs in People with dementia

The following section answers the overall research question “Which symptom assessment methods are applied by nurses to recognise holistic palliative care needs in people with dementia?”.

#### Nurse population

RNs were the biggest group of the review’s population. In many studies, only the view of RNs, regardless of their role, was explored [62, 64, 66–68, 73, 74, 79, 82, 88–90, 93, 94, 97], thus not considering important voices of other health care professionals and nursing aids, with the exception of four studies [72, 81, 83, 84].

### Different assessment methods used by nurses

**Fig. 2:**
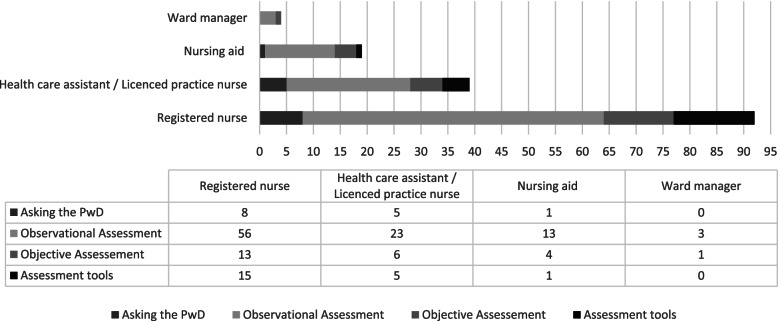
Studies reporting how nurses assess needs in People with dementia* *Multiple techniques and nurse groups in one paper possible *Count: How many studies report nurses using this technique in *n* = 37 articles

The different assessment methods used by nurses are illustrated in Fig. 2, based on the 37 studies identified for this review. Four main assessment methods emerged from the data, these are observational assessment, objective assessment, asking the People with dementia, and using standardised assessment tools.

Nurses most often used observational assessment to assess predominately pain but occasionally other needs too. Figure 2 shows in how many studies nurses selected observational assessment methods (RNs [73, 74, 76–80, 85, 87–93], followed by HCAs [72, 76, 78, 81, 84, 87, 92], Nursing aids [72, 77, 91], and Ward managers [78]). Unqualified nursing staff tend not to ask the person with dementia directly about his / her pain, even if they observe clues of pain during routine care [77]. The use of assessment tools is also less prevalent in HCAs or Nursing aids but if they used an assessment tool, the rating scales were preferred [65, 72]. Others felt assessing pain intensity unhelpful [71] or the appropriate person-centred measure was not available [96].

#### Observational assessment

Observational assessment technique means that nurses rely heavily on personal observational accounts such as behavioural changes, body movement, facial expression, verbalisation (e.g. crying), vocalisation (e.g. swearing), restlessness/agitation, and other (e.g. administering pain medication). In the following paragraph, those observational accounts are further explained:

##### Behavioural changes

In 17 qualitative- and two quantitative studies, behavioural change was the predominant cue indicating that the person with dementia was in pain [62, 63, 73–81, 84, 85, 87, 88, 90–93, 95]. In order to detect behavioural changes, nurses felt that they had to know the person with dementia [85], however this is difficult to do in the emergency unit [79]. As behavioural change can also be a symptom of the dementia deteriorating [85], nurses felt that they have to rely on their intuition and personal judgement to decide whether or not the change in behaviour is need related [85, 86, 93].

##### Body movement

Nurses in one study felt that body movement was the most important indicator for pain in people with dementia [77]. Some listed it as one assessment method within a list of assessment methods [72, 87, 91, 92], others put more emphasis on the observation of repetitive body movements [78] to recognise if the person with dementia is actively resting or guarding a body part to avoid pain [75, 83, 84, 89, 94].

##### Facial expression

Facial expression was often understood as a cue for a person with dementia being in pain [68, 72, 73, 87–90, 93]. However, nurses felt that facial expression (e.g. grimacing) could also be interpreted as a sign of needs other than pain [77].

##### Vocalisation / Verbalisation (e.g., crying)

Verbal clues are helpful for nurses assessing pain as it often occurs together with behavioural changes, thus confirming a need or care issue [68, 77, 78, 89, 93, 95]. For some nurses the expression of pain by the person with dementia is the trigger for further investigation [92]. Nurses in one study interpreted verbal behaviours as a pain indicator if these behaviours would disturb others [63]; others interpreted the volume in the person`s with dementia voice to diagnose pain [88]. Some nurses were concerned if a person with dementia repetitively vocalises needs such as pain or fatigue [78], other nurses equated such vocalisation with care seeking behaviour [78]. Vocalisation of pain by people with dementia was often described as occurring with distressed and aggressive behaviour [63, 75, 77, 87–89].

##### Restlessness/agitation

Restlessness and agitation, which may be regarded as part of neuropsychiatric symptoms, are often a sign for nurses that the person with dementia is at unease or in pain [63, 77, 86, 87]. For some nurses observing restlessness and agitation was a trigger to consider possible reasons behind this phenomenon together with peers and/or based on their professional knowledge [86]. Another considered anxiety alongside restlessness/agitation [88], which adds to the complexity of the observed, leaving nurses to ask for more educational input to reflect on reasons behind such behaviour in people with dementia [87].

##### Other

Several other types of assessment techniques were only found in single studies but offer important insights into specialist fields or roles in the assessment of experienced needs in people with dementia. In one study, administering pain medication and reviewing if the person with dementia relaxes was an assessment technique used by nurses in the emergency department when the person with dementia was admitted with hip-fracture [97]. Others described a similar process of using a “trial and error” method when introducing interventions to assess and diagnose discomfort or pain experienced by people with dementia in a nursing home [78, 91]. One study reported the importance of considering confusion as a signal of pain leaving nurses keen to learn more about other cues of pain in people with dementia [62]. Increased need of sleep was reported in one study as an indicator of pain, whilst nurses emphasised that needs can often be layered in people with dementia, hinting that another need may be more visible than pain at times [77]. Two studies described decreased appetite as a clue for nurses that the person with dementia could be in pain [77, 87]. This was only reported in the oldest record included in this review [87], but it may well be a valid and important clue. One study described environmental factors, such as weather change or cold weather, as an important factor to consider in nurse’s assessment of pain in people with dementia with arthritis, as this resident group may be more prone to pain during particular seasons [95]. Actively dying was described in a rather physiological process and nurses assumed that dying people with dementia must be more likely in pain [80].

#### Objective assessment

Among objective assessment methods were physiological cues, which nurses used in their assessment of pain. In the acute environment and in the nursing home setting, vital signs such as increased blood pressure, elevated pulse, or raised respiratory rate were important cues for nurses to become alert that the person with dementia might be in pain [64, 75, 76, 88, 91]. More obvious signs of pain (e.g. bruises, edema) were also being assessed thoroughly in various settings and by nurses with different qualifications [75, 78, 80, 81, 83]. Impaired range of motion (ROM) [75, 84, 91] or sudden decline of physical function [70, 83, 91, 92] were an important and frequent consideration for nurses in the assessment of pain. One record suggested if the pain was not confirmed after physiological assessment, including taking into account potential respiratory- or urinary tract infection, nurses concluded that the person`s with dementia pain must be of emotional origin and family would then be called immediately [75].

#### Asking the people with dementia

Communication was a vital part for nurses in the assessment of pain and other needs, mentioned as a frequently used method to elicit pain during routine care in eight qualitative studies. Some nurses tended to ask the person with dementia about pain directly in their own home and emergency department [72, 84, 97]. Communication was also practiced as the first method of assessment in acute care settings [92, 94], where metaphors communicated by people with dementia were analysed to diagnose whether or not the person with dementia was in pain [92]. Communication is often the first method used in assessing pain in hip fracture of people with dementia in a nursing home [88] and the second choice of assessment of pain in the acute care setting in people with dementia after a hip fracture [89, 90]. As the person with dementia is often unable or thought to be unable to self-report, nurses used different communication techniques to elicit an answer from the person with dementia, which they could rely on. Such techniques involved speaking directly with the person with dementia, speaking to him/her whilst holding their hand or touching his/her shoulder to feel the slightest confirmatory nod or hand squeeze or tap from him/her [89, 90].

#### Assessment tools

Overall, assessment tools for pain, including use of standard assessment tools, were reported in 19 of 37 studies. In this paragraph, most quantitative studies of this scoping review are reported for the first time because those studies often referred to nursing documentation and completion of evidence-based assessment tools in people with dementia in pain [61]. The Abbey pain scale and Pain Assessment in Advanced Dementia Scale were the most frequently mentioned assessment tools for people with dementia in pain [61, 66, 67, 71, 73, 79, 81, 93, 96]. When nurses were under time pressure, assessment tools were a welcomed method to assess pain and evaluation of pain was more likely when nurses were able to work from a completed Pain Assessment in Advanced Dementia Scale [79]. They were also used when it was an organisational requirement on admission or when nurses were participating in training to do so. Evidence based assessment with patient-reported outcome mesures or self-reported tools was found in less than 45% of all documentations in one study [61]. Nurses were using standard written assessment tools or guidelines [70, 96], but were also referring to observational assessment methods at the same time [63, 68]. The use of a pain rating scale was documented in a few studies and in a variety of settings [64–66, 68, 72, 73, 75, 84, 95] but in one record as the assessment method with the least priority [84]. Rating scales were used in the acute care environment by more than half of the registered nurses [64] and were found not to be helpful in people with advanced dementia as the question about pain intensity was left unanswered [76, 88]. In fact, in most studies, which reported the use of pain rating scales during routine care, it remained unclear how their use supported the nurses throughout the assessment process. And one study emphasised this because in over half of their nursing records reviewed (*n* = 342), nurses in the acute- and long-term setting were not using any pain assessment tools during provision of care [71].

For the assessment and diagnosis of delirium in people with dementia in the acute setting, nurses rely on altered level consciousness and fluctuation and change in the person`s with dementia cognition measured by the Confusion Assessment Method tool [69]. Behavioural symptoms were approached with a standard written assessment tool [63].

### Timing and triggers for holistic needs assessment

**Fig. 3:**
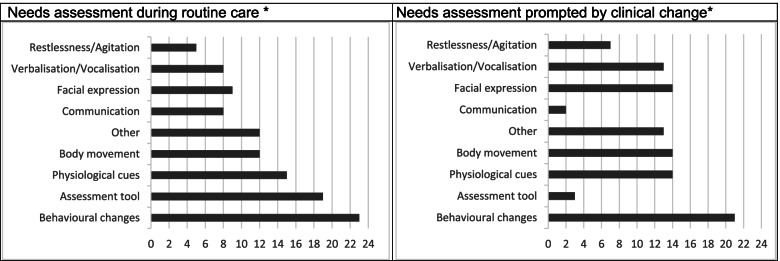
Difference between preferences of nurses’ assessment techniques during routine care and when prompted by clinical change in People with dementia *Multiple techniques and nurse groups in one paper possible *Count: How many papers report nurses using this technique in *n* = 37 articles

When results were charted, it became apparent that there are two time points when nurses assess needs and other care issues in everyday practice. These time points of assessments were either during routine care delivery or when nurses were prompted by the person with dementia to conduct an assessment unexpectedly. Figure 3 illustrates the comparison made between nurses’ assessment during routine care and assessment techniques when nurses felt triggered to conduct a needs assessment using studies included in this review.

Observational assessment methods were most commonly used when people with dementia presented with a change in their usual behaviour or showed signs of distress to assess pain in both routine needs assessment and unforeseen needs assessment [62, 63, 73–81, 84, 85, 87, 88, 90–93, 95]. However, there were differences between how nurses assessed pain in the routine assessment, versus when they were prompted by the person with dementia to do an assessment. Nurses were prompted to assess pain if the person with dementia was vocalising [65, 71, 73, 76–80, 91, 93, 96], verbalising [68, 71, 72, 77, 78, 80, 85, 88, 92, 93, 96], or grimacing [65, 68, 71, 73, 76–80, 83, 85, 96] their expression of pain and were more likely to take action based on these visible/audible cues than they were by indications presented in the routine assessment.

Surprisingly, people with dementia were rarely asked about pain when nurses felt triggered to conduct a pain assessment due to e.g., person`s with dementia general discomfort [61]. Assessment tools were also rarely used when nurses came across a person with dementia in pain unexpectedly. However, in the post-operative setting for people with dementia after hip fracture, there was a routine assessment schedule established during routine nursing care [90]. Nurses also used a person-centred outcome measure after providing therapy or administering medication when the person with dementia showed visible signs of pain [68]. In both nursing homes and acute care wards, there were two studies stating to be using formal assessments to assess pain [63, 70]. In an acute care hospital, assessment tools were used by nurses to diagnose whether or not the person with dementia was experiencing a delirious episode [69].

Only one instance of systematic pain assessment was discovered in this review. One qualitative study with a sample size of *n* = 13 nurses reported that every nurse completes a so-called “assessment cycle,” where nurses assess, intervene and evaluate the intervention by approaching each pain assessment with the same algorithm [75]. Objective and then observational assessment methods were used, but without the support of any assessment tools [75]. In two studies, however, a decision-making process was described where nurses contemplate and reflect on the observations and intelligence received about the person with dementia by peers. In doing so they grew the knowledge resources available within the team [86, 92].

#### Initial steps leading to a comprehensive assessment of needs

23 out of 37 studies included into this review captured who or where nurses tend to ask/seek for confirmation and/or verification of observed palliative care needs in people with dementia. of the need concerned.

##### Verification of pain in People with dementia

Relatives of people with dementia who were admitted to the acute care setting with pain [67, 73, 88, 92] were asked for more information about the person`s with dementia usual behaviour or his/her history of pain in order to diagnose assessed cues. One study recognised visitors, who came to see the person with dementia, as important sources of additional information [92]. Nurses in acute care environments had to find ways to gather as much information — and as quickly as possible — for the pain to be managed because they didn’t know the person with dementia [73, 88, 97]. Only one record reported acute care ward nurses asking other nurses for their opinion or knowledge or consulted referral documentation about the person with dementia [81].

Experienced nurses in looking after people with dementia, who are admitted with hip-fracture in the emergency department were often asked for their opinion to introduce pain medication rapidly [97]. This is distinct from intensive care wards, where nurses frequently called on colleagues from the ward and/or nursing home where the person with dementia was transferred from to receive more insight. They frequently inquired about the person`s with dementia pain cues in addition to information already received from relatives [89].

In hospice and home care environment relatives were easier to find [81, 82, 84], there are more nurses to consult, who can fall back on vast amount of experience in working with people with dementia [81, 82, 84], and nursing documentation is available [81] to verify pain in people with dementia. If nurses were in a consultant advisory role in nursing homes and home districts, the consultant nurse had to rely on documentation and had to be able to reach relatives for the pain assessment [82].

The largest information resource for nurses working in nursing homes and identifying pain in people with dementia were the people with dementia relatives [75, 82, 85, 86, 90, 93, 95, 96]. Paradoxically, one record reported that nurses didn’t like to ask family due to possible “hidden agendas” within the family [85]. Colleagues and peers represented an important information anchor for nurses [81, 82, 86, 90, 95] and the longer the person with dementia was admitted the less important became the available documentation because nurses became familiar with the person with dementia and were therefore less likely to refer to the nursing documentation for pain cues in their residents with dementia [81, 95]. Knowing a person with dementia well has been shown to be critical for nurses in nursing homes to determine location of pain [76, 77, 85, 93, 95]. When nurses knew the person`s with dementia communication abilities in advance, it made a difference in how accurately they could make a diagnosis of pain [90].

##### Verification of pain and other needs

When pain and other needs were present in people with dementia, knowing the person with dementia well helped nurses confirm pain in two studies [83, 87]. Exchanging information amongst nursing staff was only found to be helpful when RNs listened to HCAs observational accounts [83].

##### Verification of discomfort

Knowing the person with dementia well was an important way for nursing home nurses to distinguish discomfort from other needs [91]. It was equally as helpful as having a conversation with relatives [91].

##### Verification of behavioural symptoms

Listening to nursing assistants to verify behavioural symptoms was in one nursing home most helpful in order to confirm change in behaviour apart from consulting relatives and be guided by the nursing documentation [78].

## Discussion

To our knowledge, this is the first scoping review providing an overview about nurse’s techniques with corresponding assessment methods applied to assess holistic palliative care needs in people with dementia in clinical practice. Observational assessment methods were the preferred assessment method used by nurses, regardless of their qualifications or work settings. Family and friends were the most used resource by nurses seeking to verify subjective and objective observations of pain. If time was available, peers and the wider interdisciplinary team could offer insights that placed pain observations in the context of the people with dementia life story, living situation, and disease situation. Nurses used assessment tools in acute care environments, and environments where it was an institutional requirement. There was often no mention about which formal assessment tool was used. The most frequently used rating scale to assess pain intensity was either the numerical rating scale or the visual analogue scale but also found to be unhelpful when people with dementia were thought to be unable to self-report. Nurses felt triggered to conduct a needs assessment when behavioural symptoms were present but more prompted when pain was vocalised in comparison to other successful needs assessment techniques (e.g. communication) during routine care. There is little evidence of nurses pursuing the nursing process (assessment, diagnosis, planning, intervention, evaluation) after using an assessment method in their needs assessment to strive and improve the care provided. Nurses’ opportunity to verify pain with relatives, as well as knowing the person with dementia, are both advantages leading to better identification of pain and other symptoms. Of course, it is not only the assessment which matters, but subsequent actions to improve the symptoms identified.

Our results suggest that front-line nurses have knowledge about pain behaviour as outlined by the American Geriatrics Society in 2002 [98]. But they only followed two of five first steps of pain assessment methods in the hierarchy of pain assessment; to observe people with dementia behaviour and to ask the person with dementia to self-report [99]. To ask the person with dementia about pain and other needs requires effective communication skills, which nurses often are not confident about [100]. Additionally, asking the person with dementia about pain is perceived by nurses as time consuming thus adding to the workload pressure [28], which may be another reason why only a few nurses in our review took the time to communicate with people with dementia in unexpected assessment situations. Best possible effort must be undertaken at times of assessment to ask (verbally and non-verbal) the person with dementia about their felt needs in order to come closest to their truth as possible [101]. This means that the nurse chooses an appropriate assessment method depending on the individual’s communication abilities. The included studies didn’t report a range of assessment methods in relation to communication abilities, which becomes even more important when someone doesn’t communicate anymore. Therefore, a person-centred, proxy reported, outcome measure seems to be a good compromise because its holistic nature is implied by the person-centeredness, it is uncomplicated to complete, and can be better included into the pressurised work-environment, and/or when the person with dementia is no longer able to respond, and may help guide nurses who don’t feel confident in dementia care [102]. However, it remains proxy-reported, which means that the person with dementia may rate the items differently if they could or are asked to voice their need [103].

Person-centred care may be understood to focus on a conclusive list of needs in people with dementia [101], but Goni-Fuste et al. found in their systematic review, that evidence is fragmented about the content of the respective domains (physical, psycho-social, and spiritual) within a holistic needs assessment [104]. The authors found that the domains differ in meaning indicating that there is no holistic needs assessment fit for all life-limiting illnesses such as dementia [104].

In our review, changes in people with dementia’s behaviour seem to motivate nurses to do a needs assessment, but with almost exclusive focus on pain and not considering other symptoms, such as for example anxiety, discomfort, or depression, which may have been prevalent at that time as well [105]. Van der Steen et al. found items to elicit pain or discomfort in assessment tools overlapped between contexts [106], indicating the complexity of unpicking needs in people with dementia. Therefore, assessing pain in people with dementia is an important first step but if pain was not felt or already diagnosed, nurses should continue to discover and identify different types of “need behaviours” to unpick possible multiple needs. The nurses in our review have already the techniques to assess pain, which can be used to assess other needs. In theory, nurses are therefore prepared to isolate and consequently address multiple needs if guided by a holistic needs assessment tool rather than a single need tool.

Despite well established and holistic symptom- and needs assessment tools (e.g. RAI) [20], only RNs were referring to these for more information about the person`s with dementia pain. As explored before, this may be due to access restriction and training issues but means that other people in the nursing profession such as HCAs are not able to share their observations [20, 22, 23]. However, health care professionals, but also informal caregivers, would appreciate to have assessment tools accessible to them when assessing pain to document their observations [107]. This further supports our findings that person-centred tools (e.g., visual analogue scale and the numeric scale) were the most preferred tools by nurses – maybe because they are accessible and easy to use.

The most predominantly assessed need was pain. We found that a person with dementia had the best chance of having their pain recognised and assessed if the nurse knew him/her well and when he/she also had a family or friend advocating for them. But not all people with dementia have informal carers so in these cases nurses have “to guess” until they are familiar enough with the person with dementia. Methods in routinely assessing pain in people with dementia remained unchanged for 20 years, but nurses gained experience in caring for people with dementia, which may have made nurses more confident to rely on their intuition and personal judgement.

### Limitations

The present scoping review has several limitations. In our included studies, the stage of dementia was often not specified, which hindered categorisation of nurses’ assessment techniques into disease phases. Due to discordance between the studies’ keywords and indexing in databases, many studies were found through reference check suggesting some selection bias in the primary search strategy. We therefore intensified and strengthened the reference check with two researchers comparing results in order to include all available evidence. In three studies [61, 68, 92], results from other health care professionals were reported together with nurse’s opinion implying some participant bias. However, nurses were always the biggest group, well above two thirds of the participants, thus corresponding findings were essentially derived from nurses’ input. A pragmatic decision had to be made in categorising different types of nurses into RNs, HCAs, and nursing aids due to international name ambiguity. Charting data required a considerable amount of time because the data to chart was sometimes hidden in the text, which was more resource intensive than expected. Therefore, we agreed mutually amongst the study team, to have one researcher chart the data but with more time available, introducing a possible measurement bias. Finally, the scoping review only considered studies in the English and German languages, leaving studies published in another language excluded.

A full search for unpublished work was not conducted for the following reasons. Firstly, original research articles need to be peer reviewed, which is often not the case for grey literature [108]. Secondly, a full grey literature search is beyond the scope of the current review, and is unlikely to yield sufficiently new and reliable insights to warrant the time investment [108, 109].

## Conclusion

Our review identified that there is a scarcity of evidence with regards to chosen techniques and their corresponding methods in assessing needs other than pain in people with dementia by nurses. These techniques and methods, however, include critical and already existing nursing skills (e.g. observational methods) to identify other needs within the same conduct of needs assessment (e.g. pain). However, identification of holistic needs in people with dementia is more likely to succeed if the nurse assessing the needs is experienced in working with and caring for people with dementia, has the family to verify observations and is using a person-centred assessment or outcome measure in the assessment to underpin the nursing process.

### Implications for practice

To address the scarcity of evidence with regards to best practice recommendation in assessing needs other than pain in people with dementia it may be helpful to add a global person-centred screening tool to aid those invaluable and critical real-time observations directly at the bedside and support conversations with families to enquire or verify if those symptoms are observed. By documenting these subjective observations at the bedside and in a pro-active manner (e.g. daily or weekly depending on the setting, without waiting for symptoms to begin), then no crucial information will be lost and the nurse looking after a person with dementia will get an instant overview of a person’s needs so that interventions can be introduced rapidly in order to improve care.

Furthermore, nurses should seek to involve family, informal carers, and other professionals in the conversation to get to know the person with dementia as well as possible. Not only can the nurse then get to know the person with dementia, which is critical to assess needs, but also broaden her experience in the care of people with dementia, which will influence her future practice.

### Implications for research

Evidence about methods to recognise and assess needs other than pain in people with dementia is scarce. We were interested in how front-line nurses are assessing needs and other care issues in people with dementia and found that practice has not changed over 20 years. Nurses take behavioural changes from people with dementia as a cue for pain – but we still don’t know how other needs such as anxiety or breathlessness are assessed in a holistic approach. There is evidence that assessment tools for specific needs such as delirium and pain are in use. There is a gap in knowledge about the routine use of assessment tools for other needs than pain or delirium in people with dementia. However, we also found that nurses are demonstrating versatility in the techniques they use within the assessment methods reported. With the increasing number of people with dementia and palliative care needs, front-line nurses gain increased experience in the care of people with dementia. By channelling such nursing techniques into a holistic, person-centred assessment tool, distinct patterns for different need behaviours may start to appear. It is therefore critical, to include implementation considerations alongside introduction of a person-centred screening tool, coordinated with specific assessment tools (e.g. Neuropsychiatric assessment) if appropriate, to improve needs recognition in all settings.

Registered Nurses were the biggest group in this review, but it is important to highlight that HCAs and other care staff can also provide expert opinion on the people they are caring for and have excellent observational skills to recognise and assess needs. Future studies should therefore be more inclusive of HCAs and other care staff to gain more insight into needs assessment, which supports to meet holistic palliative care needs in people with dementia.

## Supplementary Information

**Additional file 1.**

## Data Availability

All data generated or analysed during this study are included in this published article [and its supplementary information files].

## References

[1] Sleeman KE, de Brito M, Etkind S, et al. The escalating global burden of serious health-related suffering: projections to 2060 by world regions, age groups, and health conditions. Lancet Glob Health. 2019;7(7):E883–92. 10.1016/S2214-109X(19)30172-X PMC6560023 31129125

[2] Abreu W, Tolson D, Jackson GA, Staines H, Costa N (2019). The relationship between frailty, functional dependence, and healthcare needs among community-dwelling people with moderate to severe dementia. Health Soc Care Community.

[3] Mason B, Nanton V, Epiphaniou E, 'My body's falling apart' (2016). Understanding the experiences of patients with advanced multimorbidity to improve care: serial interviews with patients and carers. BMJ Support Palliat Care.

[4] Livingston G, Huntley J, Sommerlad A (2020). Dementia prevention, intervention, and care: 2020 report of the <em>Lancet</em> Commission. Lancet.

[5] (WHO) WHO. WHO Defintion of Palliative Care. 2017; http://www.who.int/cancer/palliative/definition/en/.

[6] Murray SA, Kendall M, Mitchell G, Moine S, Amblàs-Novellas J, Boyd K. Palliative care from diagnosis to death. BMJ. 2017;356:j878. 10.1136/bmj.j878 28242747

[7] Parikh RB, Bowman B, Dahlin C, Twohig JS, Meier DE (2017). Scalable principles of community-based high-value care for seriously ill individuals: diamonds in the rough. Healthcare.

[8] Pask S, Pinto C, Bristowe K (2018). A framework for complexity in palliative care: a qualitative study with patients, family carers and professionals. Palliat Med.

[9] Shiells K, Pivodic L, Holmerová I, Van den Block L. Self-reported needs and experiences of people with dementia living in nursing homes: a scoping review. Aging Mental Health. 2020;24(10):1553–68. 10.1080/13607863.2019.1625303 31163987

[10] Harding AJE, Morbey H, Ahmed F (2019). What is important to people living with dementia?: the 'long-list' of outcome items in the development of a core outcome set for use in the evaluation of non-pharmacological community-based health and social care interventions. BMC Geriatr.

[11] Cadieux MA, Garcia LJ, Patrick J (2013). Needs of people with dementia in long-term care: a systematic review. Am J Alzheimers Dis Other Demen.

[12] Wolverson E, Moniz-Cook E, Dunn R, Dunning R. Family carer perspectives on the language of behaviour change in dementia: an online mixed methods survey. Age Ageing. 2022;51(3):afac047. 10.1093/ageing/afac047. 10.1093/ageing/afac047 35231095

[13] van Dalen-Kok AH, Pieper MJ, de Waal MW, Lukas A, Husebo BS, Achterberg WP (2015). Association between pain, neuropsychiatric symptoms, and physical function in dementia: a systematic review and meta-analysis. BMC Geriatr.

[14] Sekse RJT, Hunskår I, Ellingsen S (2018). The nurse's role in palliative care: a qualitative meta-synthesis. J Clin Nurs.

[15] National Institute for Health Research (NIHR) (2015). James Lind Alliance Priority Setting Partnerships; Palliative and end of life care Top 10.

[16] Mistry B, Bainbridge D, Bryant D, Tan Toyofuku S, Seow H (2015). What matters most for end-of-life care? Perspectives from community-based palliative care providers and administrators. BMJ Open.

[17] Giménez-Llort L, Bernal ML, Docking R, et al. Pain in older adults with dementia: a survey in Spain. Front Neurol. 2020;11:592366. 10.3389/fneur.2020.592366. 10.3389/fneur.2020.592366 PMC7715009 33329344

[18] While C, Jocelyn A (2009). Observational pain assessment scales for people with dementia: a review. Br J Community Nurs.

[19] National Cancer Action Team. Holistic common assessment of supportive and palliative care needs for adults requiring end of life care. In: program National end of life care program. United Kingdom: National Health Service (NHS); 2010.

[20] interRAI. Home Care (HC). 2018; http://www.interrai.org/home-care.html.

[21] Morris JN, Fries BE, Steel K (1997). Comprehensive clinical assessment in community setting: applicability of the MDS-HC. J Am Geriatr Soc.

[22] Conroy SP, Bardsley M, Smith P (2019). Health Services and Delivery Research. Comprehensive geriatric assessment for frail older people in acute hospitals: the HoW-CGA mixed-methods study.

[23] Koppitz A, Bosshard G, Blanc G, Hediger H, Payne S, Volken T (2017). Pain Intervention for people with Dementia in nursing homes (PID): study protocol for a quasi-experimental nurse intervention. BMC Palliat Care.

[24] Bausewein C DB, Currow DC, Downing J, Deliens L, Radbruch L, et al. EAPC White paper on Outcome Measurement in Palliative Care: improving practice, attaining outcomes and delivering quality services. Recommendations from the European Association for Palliative care (EAPC) Task Force on outcome measurement. Palliat Med. 2016;30(1):6–22. 10.1177/0269216315589898 26068193

[25] Donabedian A. The definition of quality and approaches to its assesment. Vol 1. Ann Arbor: Health Administration Press; 1980.

[26] Laging B, Kenny A, Bauer M, Nay R (2018). Recognition and assessment of resident' deterioration in the nursing home setting: A critical ethnography. J Clin Nurs.

[27] van der Steen JT, Radbruch L, Hertogh CM (2014). White paper defining optimal palliative care in older people with dementia: a Delphi study and recommendations from the European association for palliative care. Palliat Med.

[28] Burns M, McIlfatrick S (2015). Palliative care in dementia: literature review of nurses' knowledge and attitudes towards pain assessment. Int J Palliat Nurs.

[29] Tsai IP, Jeong SY, Hunter S (2018). Pain assessment and management for older patients with dementia in hospitals: an integrative literature review. Pain Manag Nurs.

[30] Higginson IJ, Hart S, Koffman J, Selman L, Harding R (2007). Needs assessments in palliative care: an appraisal of definitions and approaches used. J Pain Symptom Manage.

[31] Bradshaw J (1972). Taxonomy of social need.

[32] Aveyard H, Payne SA, Preston NJ. A post-graduate's guide to doing a literature review in health and social care. Oxford: Oxford University Press; 2016.

[33] Guides VLR. How to conduct a literature review (Health Sciences). 2018; https://guides.library.vcu.edu/health-sciences-lit-review/question#s-lg-box-9982939.

[34] Hui D, Bruera E (2017). The Edmonton symptom assessment system 25 years later: past, present, and future developments. J Pain Symptom Manage.

[35] Daly WM, Carnwell R (2003). Nursing roles and levels of practice: a framework for differentiating between elementary, specialist and advancing nursing practice. J Clin Nurs.

[36] Konetzka RT, Lasater KB, Norton EC, Werner RM (2018). Are recessions good for staffing in nursing homes?. Am J Health Econ.

[37] Imhof L, Rüesch P, Schaffert R, Mahrer-Imhof R, Fringer A, Kerker-Specker C (2011). Perspektiven in der professionellen Pflege in der Schweiz: Literaturgestützte Analyse zukünftiger Entwicklungstendenzen.

[38] Griffiths P, Ball J, Drennan J, Nurse staffing and patient outcomes: Strengths and limitations of the evidence to inform policy and practice (2016). A review and discussion paper based on evidence reviewed for the national institute for health and care excellence safe staffing guideline development. Int J Nurs Stud.

[39] Royal College of Nursing (RCN). Core Competences: Integrated core career and competence framework for registered nurses. London: 2009. https://nipec.hscni.net/download/217/reading/1019/rcn-core-career-and-competence-framework-rn.pdf.

[40] Eckerblad J, Theander K, Ekdahl AW, Jaarsma T (2016). Symptom trajectory and symptom burden in older people with multimorbidity, secondary outcome from the RCT AGe-FIT study. J Adv Nurs.

[41] Dodd M, Janson S, Facione N (2001). Advancing the science of symptom management. J Adv Nurs.

[42] Sidani S. Symptom management. In: Doran DM, editor. Nursing Outcomes; The state of the Science. 2nd Edition. Sudbury: Jones & Bartlett Learning; 2011. p. 131–99.

[43] Centre for Reviews and Dissemination (CRD). International prospective register of systematic reviews (PROSPERO). 2018; https://www.crd.york.ac.uk/prospero/, 2018.

[44] McDonald A-L, Langford IH, Boldero N (1997). The future of community nursing in the United Kingdom: district nursing, health visiting and school nursing. J Adv Nurs.

[45] Schweizerische Sanitätsdirektorenkonferenz. Diplomausbildungen im Gesundheitsbereicht - ausgewählte Daten und Fakten. 2003. https://silo.tips/downloadFile/diplomausbildungen-im-gesundheitsbereich-ausgewhlte-daten-und-fakten.

[46] Quinn FM. The principles and practice of nurse education. Cheltenham: Nelson Thornes; 2000.

[47] Achterberg WP, Pieper MJC, van Dalen-Kok AH (2013). Pain management in patients with dementia. Clin Interv Aging.

[48] Brown D (2004). A literature review exploring how healthcare professionals contribute to the assessment and control of postoperative pain in older people. J Clin Nurs.

[49] Chalmers JM, Pearson A (2005). A systematic review of oral health assessment by nurses and carers for residents with dementia in residential care facilities. Spec Care Dentist.

[50] Chandler RC, Zwakhalen SMG, Docking R, Bruneau B, Schofield P (2017). Attitudinal & knowledge barriers towards effective pain assessment & management in dementia: A narrative synthesis. Curr Alzheimer Res..

[51] Cowan DT, Fitzpatrick JM, Roberts JD, While AE, Baldwin J (2003). The assessment and management of pain among older people in care homes: current status and future directions. Int J Nurs Stud.

[52] Curtiss CP (2010). Challenges in pain assessment in cognitively intact and cognitively impaired older adults with cancer. Oncol Nurs Forum.

[53] Davies E, Male M, Reimer V, Turner M, Wylie K (2004). Pain assessment and cognitive impairment: part 1. Nurs Stand.

[54] Gregory J (2015). The complexity of pain assessment in older people. Nurs Older People.

[55] Miller LL, Talerico KA (2002). Pain in older adults. Annu Rev Nurs Res.

[56] Soscia J (2003). Assessing pain in cognitively impaired older adults with cancer. Clin J Oncol Nurs.

[57] Webster J (2011). Improving care for people with dementia in acute hospital: the role of person-centred assessment. Qual Ageing Older Adults.

[58] Tricco AC, Lillie E, Zarin W (2018). PRISMA Extension for Scoping Reviews (PRISMA-ScR): checklist and explanation. Ann Intern Med.

[59] Dunphy LM, Winland-Brown J, Porter B, Thomas D. Primary care: art and science of advanced practice nursing. Philadelphia: FA Davis; 2015.

[60] Koppitz A, Bosshard G, Blanc G, Hediger H, Payne S, Volken T (2017). Pain Intervention for people with Dementia in nursing homes (PID): study protocol for a quasi-experimental nurse intervention. BMC Palliat Care.

[61] Andrews SM, Dipnall JF, Tichawangana R (2019). An exploration of pain documentation for people living with dementia in aged care services. Pain Manag Nurs.

[62] Barry HE, Parsons C, Peter Passmore A, Hughes CM (2012). An exploration of nursing home managers' knowledge of and attitudes towards the management of pain in residents with dementia. Int J Geriatr Psychiatry.

[63] Cohen-Mansfield J, Jensen B, Resnick B, Norris M (2012). Assessment and treatment of behavior problems in dementia in nursing home residents: a comparison of the approaches of physicians, psychologists, and nurse practitioners. Int J Geriatr Psychiatry.

[64] Herr K, Titler MG, Schilling ML (2004). Evidence-based assessment of acute pain in older adults: current nursing practices and perceived barriers. Clin J Pain.

[65] Mezinskis PM, Keller AW, Luggen AS (2004). GN management: assessment of pain in the cognitively impaired older adult in long-term care [corrected] [published erratum appears in GERIATR NURS 2004 May-Jun;25(3):191]. Geriatr Nurs.

[66] Minaya-Freire A, Subirana-Casacuberta M, Puigoriol-Juvanteny E, Ramon-Aribau A (2021). Pain management nursing practice assessment in older adults with dementia. Nurs Open.

[67] Muñoz-Narbona L, Cabrera-Jaime S, Lluch-Canut T, Castaño PB, Roldán-Merino J (2020). E-Learning course for nurses on pain assessment in patients unable to self-report. Nurs Educ Pract.

[68] Sirsch E, Zwakhalen S, Gnass I (2015). Schmerzassessment und Demenz - Deutschsprachige Ergebnisse eines europäischen surveys. Pflege Gesellschaft.

[69] Steis MR. Nurse recognition of delirium superimposed on dementia in hospitalized older adults. Pennsylvania State University; 2009.

[70] Timmons S, O'Shea E, O'Neill D (2016). Acute hospital dementia care: results from a national audit. BMC Geriatr.

[71] Zwakhalen S, Docking RE, Gnass I (2018). Pain in older adults with dementia : a survey across Europe on current practices, use of assessment tools, guidelines and policies. Der Schmerz.

[72] Blomqvist K, Hallberg IR (2001). Recognising pain in older adults living in sheltered accommodation: the views of nurses and older adults. Int J Nurs Stud.

[73] Brorson H, Plymoth H, Örmon K, Bolmsjö I (2014). Pain relief at the end of life: nurses' experiences regarding end-of-life pain relief in patients with dementia. Pain Manag Nurs.

[74] Chang E, Daly J, Johnson A (2009). Challenges for professional care of advanced dementia. Int J Nurs Pract.

[75] Chang SO, Oh Y, Park EY, Kim GM, Kil SY (2011). Concept analysis of nurses' identification of pain in demented patients in a nursing home: development of a hybrid model. Pain Manag Nurs.

[76] Clark L, Jones K, Pennington K (2004). Pain assessment practices with nursing home residents. West J Nurs Res.

[77] Closs SJ, Cash K, Barr B, Briggs M (2005). Cues for the identification of pain in nursing home residents. Int J Nurs Stud.

[78] Cohen-Mansfield JPD, Creedon MPD (2002). Nursing staff members' perceptions of pain indicators in persons with severe dementia. Clin J Pain Jan/Feb.

[79] Fry M, Arendts G, Chenoweth L (2017). Emergency nurses' evaluation of observational pain assessment tools for older people with cognitive impairment. J Clin Nurs (John Wiley & Sons, Inc.).

[80] Gilmore-Bykovskyi AL, Bowers BJ (2013). Understanding nurses' decisions to treat pain in nursing home residents with dementia. Res Gerontol Nurs.

[81] Jansen BW, Brazil K, Passmore P (2017). Exploring healthcare assistants' role and experience in pain assessment and management for people with advanced dementia towards the end of life: a qualitative study. BMC Palliat Care.

[82] Karlsson C, Sidenvall B, Bergh I, Ernsth-Bravell M (2012). Registered nurses´ view of performing pain assessment among persons with dementia as consultant advisors. Open Nurs J.

[83] Karlsson C, Sidenvall B, Bergh I, Ernsth-Bravell M (2013). Certified nursing assistants' perception of pain in people with dementia: a hermeneutic enquiry in dementia care practice. J Clin Nurs.

[84] Karlsson CE, Bravell ME, Ek K, Bergh I (2015). Home healthcare teams' assessments of pain in care recipients living with dementia: A Swedish exploratory study. Int J Older People Nurs.

[85] Kenefick AL (2004). Caring for Cognitively Impaired Nursing Home Residents with Pain. Int J Hum Caring..

[86] Koppitz A, Bosshard G, Kipfer S, Imhof L (2016). Decision-making in caring for people with dementia at the end of life in nursing homes. Int J Palliat Nurs.

[87] Kovach CR, Griffie J, Muchka S, Noonan PE, Weissman DE (2000). Nurses' perceptions of pain assessment and treatment in the cognitively impaired elderly: it's not a guessing game. Clin Nurs Specialist.

[88] Krupic F, Biscevic M, Spahic E (2020). Experience of nurses in assessing postoperative pain in hip fracture patients suffering from dementia in nursing homes. Med.

[89] Krupic F, Grbic K, Senorski EH, Lepara O, Fatahi N, Svantesson E (2020). Experience of intensive care nurses in assessment of postoperative pain in patients with hip fracture and dementia. Materia Sociomedica.

[90] Krupic F, Sadic S, Seffo N (2018). Experience of registered nurses in assessing postoperative pain in hip fracture patients with dementia. Med.

[91] Lee SJ, Park MS, Choi YR, Chang SO (2020). Concept Development of Identification of Discomfort for Nursing Home Patients With Advanced Dementia. Int J Nurs Knowl..

[92] Lichtner V, Dowding D, Allcock N (2016). The assessment and management of pain in patients with dementia in hospital settings: a multi-case exploratory study from a decision making perspective. BMC Health Serv Res.

[93] Lundin E, Godskesen TE (2021). End-of-life care for people with advanced dementia and pain: a qualitative study in Swedish nursing homes. BMC Nurs.

[94] Manias E (2012). Complexities of pain assessment and management in hospitalised older people: a qualitative observation and interview study. Int J Nurs Stud.

[95] Monroe TB, Parish A, Mion LC (2015). Decision factors nurses use to assess pain in nursing home residents with dementia. Arch Psychiatr Nurs.

[96] Peisah C, Weaver J, Wong L, Strukovski J-A (2014). Silent and suffering: a pilot study exploring gaps between theory and practice in pain management for people with severe dementia in residential aged care facilities. Clin Interv Aging.

[97] Seffo N, HamrinSenorski E, Westin O, Svantesson E, Krupic F (2020). Communication and assessment of pain in hip fracture patients with dementia - experiences of healthcare professionals at an accident and emergency department in Sweden. Med.

[98] AGS Panel on Persistent Pain in Older Persons (2002). The management of persistent pain in older persons. J Am Geriatr Soc..

[99] Herr K, Coyne PJ, Ely E, Gélinas C, Manworren RCB (2019). Pain assessment in the patient unable to self-report: clinical practice recommendations in support of the ASPMN 2019 position statement. Pain Manag Nurs.

[100] Evripidou M, Charalambous A, Middleton N, Papastavrou E (2019). Nurses' knowledge and attitudes about dementia care: Systematic literature review. Perspect Psychiatr Care.

[101] Shuman SB, Hughes S, Wiener J, Gould E. Research on care needs and supportive approaches for persons with dementia. Background Paper. Research Summit on Dementia Care: Building Evidence for Services and Supports. Washington: 2017.

[102] Ellis-Smith C, Evans CJ, Murtagh FE (2017). Development of a caregiver-reported measure to support systematic assessment of people with dementia in long-term care: the integrated palliative care outcome scale for dementia. Palliat Med.

[103] Griffiths AW, Smith SJ, Martin A, Meads D, Kelley R, Surr CA (2020). Exploring self-report and proxy-report quality-of-life measures for people living with dementia in care homes. Qual Life Res.

[104] Goni-Fuste B, Crespo I, Monforte-Royo C, Porta-Sales J, Balaguer A, Pergolizzi D (2021). What defines the comprehensive assessment of needs in palliative care? An integrative systematic review. Palliative Med.

[105] Kroenke K, Gao S, Mosesso KM, et al. Prevalence and predictors of symptoms in persons with advanced dementia living in the community. J Palliative Med. 2022 [ahead of print]. 10.1089/jpm.2021.0402. 10.1089/jpm.2021.0402 PMC9492904 35357951

[106] van der Steen JT, Sampson EL, Van den Block L (2015). Tools to assess pain or lack of comfort in dementia: a content analysis. J Pain Symptom Manage.

[107] Kolanowski A, Van Haitsma K, Penrod J, Hill N, Yevchak A (2015). Wish we would have known that! communication breakdown impedes person-centered care. Gerontologist.

[108] Mahood Q, Eerd DV, Irvin E (2014). Searching for grey literature for systematic reviews: challenges and benefits. Res Synthesis Methods.

[109] Paez A (2017). Gray literature: an important resource in systematic reviews. J Evid Based Med.

