# Supplementary material for: Are nurse`s needs assessment methods robust enough to recognise palliative care needs in people with dementia? A scoping review
Source: BMC Nurs. 2022 Jul 20;21:194. doi: 10.1186/s12912-022-00947-6 (PMC9297617; doi:10.1186/s12912-022-00947-6)
Supplement: Supplementary file 1 — Additional file 1. [file 12912_2022_947_MOESM1_ESM.docx]

**Supplementary File:**

**Search String used in MEDLINE(R) via OVID**

CONCEPT 1 (OR)

Exp Nurses/
Exp Nursing Staff/
Exp Faculty, nursing/
Nurse*.mp
Nursing staff.mp
Registered nurs*.mp
Nurse practitioner*.mp
Faculty nurs*.mp
(nurse adj1 (practitioner or advanced)).mp.
((registered or staff or auxiliary) adj3 nurse*).mp.

AND

CONCEPT 2 (OR)

Exp Dementia/
Exp Frontotemporal Dementia/
Exp Cognition Disorders/
Exp Memory Disorders/
Dementia.mp
Frontotemporal Dementia.mp
Cognition Disorders.mp
Dementia patient*.mp
Cognitive impairment.mp
Cognitive dysfunction.mp
Memory disorder*.mp
Memory loss.mp

AND

CONCEPT 3 (OR)

Exp Nursing Process/
Exp Outcome and Process Assessment, Health Care/
Exp Symptom Assessment/
Exp Geriatric Assessment/
Exp Needs Assessment/
Exp Patient Handoff/
Exp Patient care planning/
Exp Nursing records/
Exp Medical Record Systems, Computerized/
Exp Nursing Audit/
Exp Nonverbal communication/
Nursing process.mp
Nursing diagnoses.mp
Nursing assessment.mp
Patient assessment*.mp
Outcome assessment.mp
Process assessment.mp
Symptom Assessment.mp
Geriatric assessment.mp
Needs assessment.mp
Handoff*.mp
Hand off*.mp
Handover*.mp
Hand over*.mp
assess* tool*.mp.
assess* method*.mp.
assessing.mp.
Decision-making.mp
Nursing documentation.mp
Patient care planning.mp
Nursing record*.mp
Shift report*.mp
Geriatric*.mp
Audit.mp
Nonverbal communication.mp
(observ* adj5 assess*).mp.

LIMITS

German and English language

Published from 2000 to present
